# Performance Indices in Wheat Chlorophyll *a* Fluorescence and Protein Quality Influenced by FHB

**DOI:** 10.3390/pathogens6040059

**Published:** 2017-11-20

**Authors:** Valentina Spanic, Marija Viljevac Vuletic, Georg Drezner, Zvonimir Zdunic, Daniela Horvat

**Affiliations:** 1 Department of Small Cereal Crops, Agricultural Institute Osijek, Juzno predgradje 17, 31000 Osijek, Croatia; georg.drezner@poljinos.hr; 2 Agrochemical laboratory, Agricultural Institute Osijek, Osijek, Juzno predgradje 17, 31000 Osijek, Croatia; marija.viljevac@poljinos.hr (M.V.V.); daniela.horvat@poljinos.hr (D.H.); 3 Department of Maize, Agricultural Institute Osijek, Osijek, Juzno predgradje 17, 31000 Osijek, Croatia; zvonimir.zdunic@poljinos.hr

**Keywords:** end-use quality, *Fusarium*, photosynthesis, test weight, *Triticum aestivum* L., 1000 kernel weight

## Abstract

Very little is known about the physiological interactions between wheat quality and Fusarium head blight (FHB), which substantially reduces wheat grain yield and quality worldwide. In order to investigate stress-induced changes in flag leaves from plants artificially inoculated with *Fusarium*, we screened for chlorophyll *a* fluorescence transient at 1, 2, 4, 7 and 14 days after *Fusarium* inoculation. Our results indicate that the maximum quantum yield of photochemistry (F_v_/F_m_) and the performance index (PI) were not affected by FHB, but there were significant differences in those two traits between different varieties and measurement times. FHB caused a significant reduction in the percentage of glutenins (GLU), high-molecular-weight (HMW), and low-molecular-weight (LMW) subunits in ‘Kraljica’ and ‘Golubica’, unlike ‘Vulkan’, where the percentage of GLU increased.

## 1. Introduction

Wheat (*Triticum aestivum* L.) is a major cereal crop grown in temperate climate areas. During crop production, both abiotic and biotic stresses occur, often acting in combinations under field conditions [1]. Climate changes can decrease the effectiveness of some resistance genes in wheat and potentially increase sensitivity to pathogens. Among the most important diseases in wheat that significantly reduce wheat production are those caused by *Fusarium* species. Fusarium head blight (FHB), also called ear blight or scab, is one of the most devastating fungal diseases of wheat and other small grain cereals and has caused serious epidemics worldwide [2]. FHB causes premature death of spikelets [3], which later become covered with pink masses of themycelium. FHB infects spike tissue and affects grain development due to inhibited photosynthesis, thus reducing yield, quality, and feeding value of the grain. Grains can be contaminated with toxins produced by *Fusarium* pathogens that are harmful to humans and livestock [4]. Because of the complex nature of the host/pathogen/environment interactions, it is difficult to control FHB, and screening for biotic stress is a rather difficult and time-consuming process. Currently, no fungicides can completely control FHB [5]. Wheat variety is one of the most important factors influencing FHB resistance, grain yield, and quality parameters [6]. Favorable weather conditions also play a significant role in the spread of this disease [7].

Physiological traits can be a powerful tool for tracing varieties conferring disease resistance. Among the relevant physiological processes, photosynthesis is the one primarily affected by foliar diseases [8], but the influence of ear pathogens on leaf photosynthesis has not yet been investigated. Chlorophyll *a* fluorescence is a very sensitive probe of the physiological status of leaves, and its performance varies in a wide range of situations [9]. The infection of plant tissue with fungal pathogens is closely linked to changes in metabolic pathways, such as photosynthesis [10]. In tomato plants infected with *F. oxysporum*, disease index was correlated with photosynthetic activity [11]. Photosynthesis in the wheat flag leaves should be the most important source of dry matter production directly influencing grain yield [12]. Up to 75% of reduced nitrogen, taken from chloroplast proteins (mainly from Rubisco), translocate to the grain [13].

FHB can cause grain quality reduction as a result of contamination by trichothecene mycotoxins produced by *Fusarium* species [14]. Wheat gluten proteins are very important because they are related to many processing properties. The end-use quality is determined by gliadins (GLI) and glutenins (GLU). GLI (ω-, α- γ-) and GLU (high-molecular-weight (HMW) glutenin subunits and low-molecular-weight (LMW) glutenin subunits) are major components of gluten, and these fractions can be considerably degraded under Fusarium infection [15,16,17]. HMW glutenin subunits play a very important role in determining genotypic variation in the bread making quality of wheat [18] and their composition has predictive value in quality analysis. Albumins and globulins (AG) are non-gluten proteins; they do not have a crucial role in defining bread making quality, but do have some influence [19].

Few studies have been conducted about the influence of FHB on wheat photosynthetic parameters. The objective of this study was to evaluate the applicability of the parameters derived from the fast chlorophyll *a* fluorescence kinetics to evaluate biotic stress response caused by *Fusarium gramineraum* and *F. culmorum* infection and distinguish disease tolerance among the tested wheat varieties and between the most important agronomical and technological properties. Additionally, we wanted to check how photosynthetic capacity in diseased heads will influence assimilation during seed filling and grain quality. Photosynthesis-associated parameters, PI and F_v_/F_m_, were determined in order to explore whether there is a possibility of using chlorophyll fluorescence as an indicator of *Fusarium* resistance for quality traits.

## 2. Results

**Figure 1:**
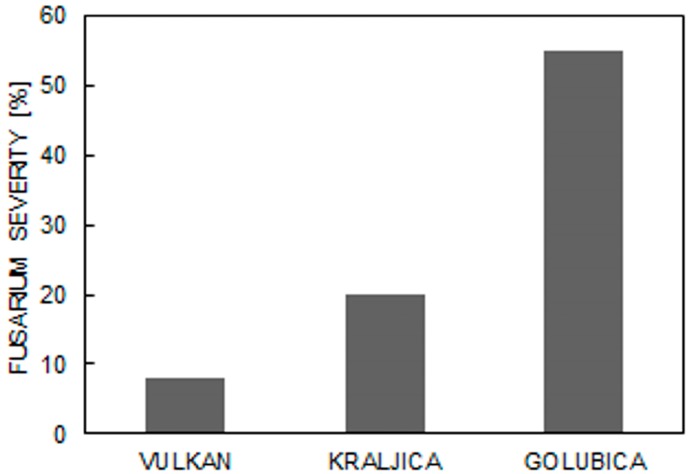
*Fusarium* severity in Fusarium head blight (FHB) treatment in three wheat varieties.

FHB severity 26 days after inoculation, compared to the ‘Golubica’ variety (55%), was lower in the ‘Vulkan’ (8%) and ‘Kraljica’ (20%) varieties (Figure 1). An ANOVA revealed that the varieties and treatments, as well as their interactions, were significant for test weight, 1000 kernel weight, % of AG, % of GLI, % of GLU, % of HMW glutenin subunits, and % of LMW glutenin subunits—except for % of AG between treatments (Table 1). Additionally, significant differences were found between varieties and measurement times for PI and F_v_/F_m_ and between variety–measurement time interactions for PI. There were no significant differences between treatments for PI, F_v_/F_m_, and interactions between varieties, measurement times, and treatments, except for PI in the variety–measurement time interaction (Table 2). Generally, under the control and FHB treatment conditions, the highest PI was observed in ‘Kraljica’ (4.22) under the FHB treatment conditions, and, in the same treatment, the lowest value was found in ‘Vulkan’ (3.42) (Table 3).

**Figure 2:**
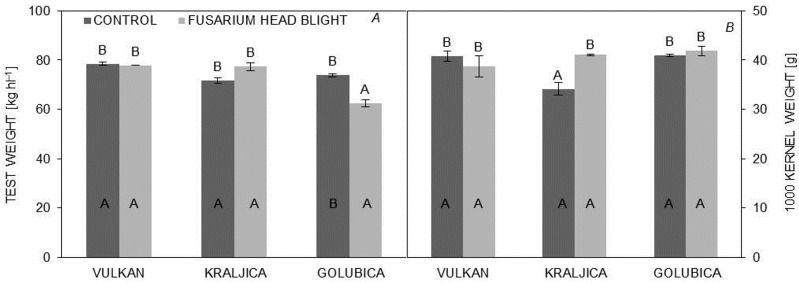
(**A**,**B**) Test weight and 1000 kernel weight in untreated and treated plants of three wheat varieties. Values are the means of two replications ± SE. Letters above the graphs indicate significantly different values (*p* < 0.05) among different varieties under the same treatment. Letters within graphs indicate significantly different values in different treatments (control and inoculation).

The ‘Golubica’ variety, compared to other varieties, was significantly decreased in test weight under FHB treatment (62.45 kg hl^−1^) compared to the control treatment. For ‘Vulkan’ and ‘Kraljica’, test weight was not statistically significant between treatments, but infected ears of ‘Kraljica’ (34.10 g) with control treatment, compared to the other two varieties, showed a significant decrease in 1000 kernel weight. Comparing the 1000 kernel weight of all varieties of FHB-infected plants with control treatment, no significant difference was found (Figure 2A,B).

**Figure 3:**
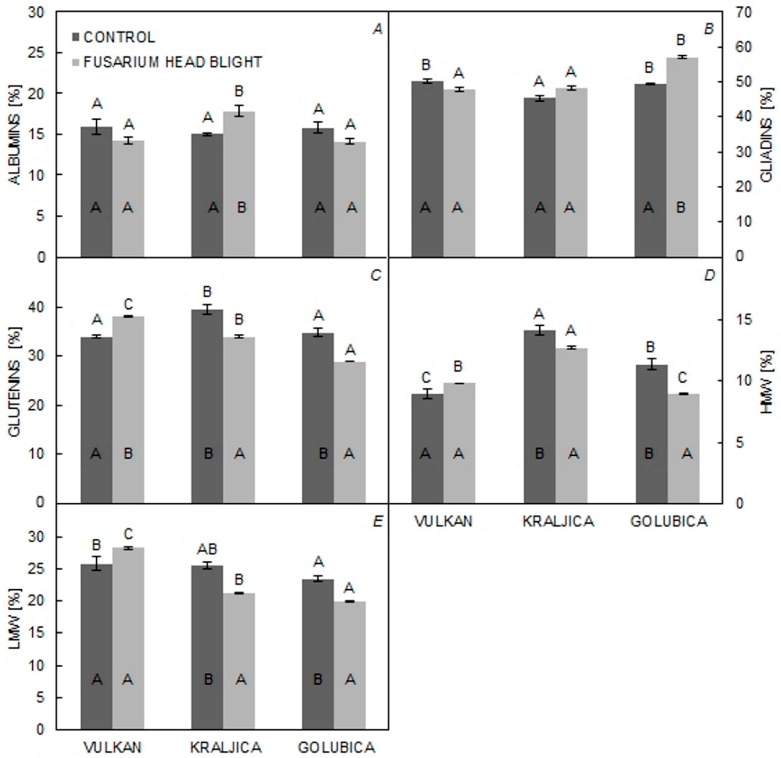
(**A**–**E**) % of AG, % of GLI, % of GLU, % of HMW and % of LMW in untreated and treated plants of three wheat varieties. Values are means of two replications ± SE. Letters under the graphs indicate significantly different values (*p* < 0.05) among different varieties under the same treatment. Letters within graphs indicate significantly different values in different treatments (control and inoculation).

The percentage of AG was significantly increased in ‘Kraljica’ with FHB treatment compared to the control treatment. The percentage of GLI was significantly increased in ‘Golubica’ in the FHB treatment group in comparison to the control group. In the control group, ‘Kraljica’ had a significantly lower percentage of GLI compared to other varieties; in contrast, both ‘Vulkan’ and ‘Kraljica’, in comparison to ‘Golubica’, had a significantly lower percentage of GLI under FHB treatment (Figure 2A,B). In the control group, ‘Kraljica’, in comparison to ‘Vulkan’ and ‘Golubica’, had a significantly higher percentage of GLU and HMW. In the FHB treatment group, compared to the control group, ‘Vulkan’ was significantly increased in the percentage of GLU; ‘Kraljica’ and ‘Golubica’ saw a significant decrease in this parameter (Figure 3C,D). The percentage of HMW was significantly lower in ‘Vulkan’, in comparison to ‘Kraljica’ and ‘Golubica’, in the control group. In ‘Kraljica’ and ‘Golubica’, the percentage of HMW and LMW was significantly lower in inoculated plants in the FHB treatment group. ‘Vulkan’, compared with the other two varieties, showed the highest percentage of LMW in the FHB treatment group (Figure 3E).

**Figure 4:**
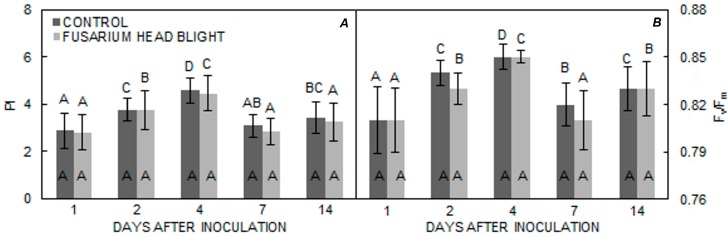
(**A**,**B**) Means ± SE for parameters PI and F_v_/F_m_ from FHB and control treatments for ‘Vulkan’ obtained at different measuring times. Letters under the graphs indicate significantly different values (*p* < 0.05) among different measuring times under the same treatment. Letters within the graphs indicate significantly different values between different treatments at one measuring point.

**Figure 5:**
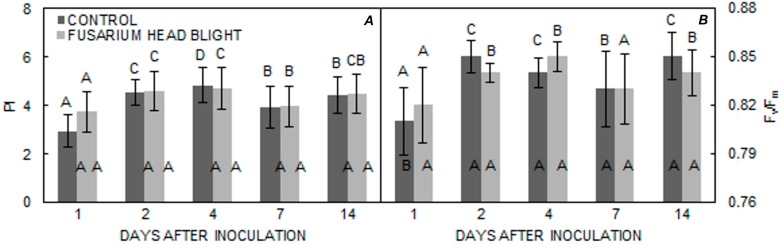
(**A**,**B**) Means ± SE for parameters PI and F_v_/F_m_ from FHB and control treatments for ‘Kraljica’ obtained at different measuring times. Letters under the graphs indicate significantly different values (*p* < 0.05) among different measuring times under the same treatment. Letters within the graphs indicate gnificantly different values between different treatments at one measuring point.

**Figure 6:**
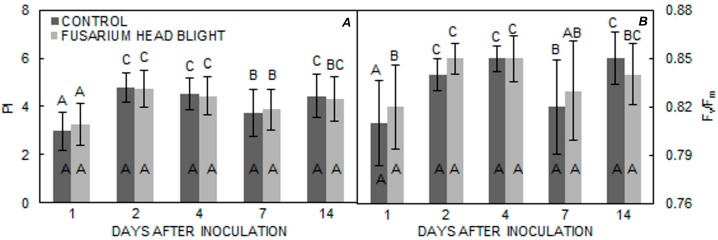
(**A**,**B**). Means ± SE for parameters PI and F_v_/F_m_ from FHB and control treatments for ‘Golubica’ obtained at different measuring times. Letters under the graphs indicate significantly different values (*p* < 0.05) among different measuring times under the same treatment. Letters within the graphs indicate significantly different values between different treatments at one measuring point.

As regards different measurement times, differences in PI and F_v_/F_m_ became statistically significant 2 days after inoculation in all three varieties in both treatments. In both groups, PI increased in all varieties until 4 days after inoculation; however, in the ‘Vulkan’ (Figure 4A) variety, PI significantly decreased in both treatments, whereas, for the ‘Kraljica’ (Figure 5A) variety, PI only significantly decreased in the FHB treatment group. In ‘Golubica’ (Figure 6A) PI was also significantly decreased in both treatments. In ‘Golubica’, PI remained unchanged 4 days after inoculation and decreased 7 days after inoculation in both treatments; in the control group, PI increased 14 days after inoculation (Figure 5A). A very similar pattern of F_v_/F_m_ was obtained in all three tested varieties. The first significant increase of F_v_/F_m_ in both treatments in all three varieties was recorded 2 days after inoculation, but was followed by a significant increase 4 days after inoculation only in ‘Vulkan’ (Figure 4B). A significant diminution in F_v_/F_m_ was evident 7 days after inoculation for all three varieties in both treatments. Fourteen days after inoculation, another increment in F_v_/F_m_ was noted in both treatments in all varieties; however, in the control group, this increment was only found in ‘Golubica’ (Figure 6B).

## 3. Discussion

The potential leaf photosynthesis and maximal crop yield are in a highly positive correlation [20]. This also indicates that photosynthesis at the single-leaf level can be an important factor for potential biomass production. However, according to Ewans and Rawson (1970) [21], ear photosynthesis contributed to grain requirements during grain development by 20–33%. The rate of photosynthesis in the flag leaves varied in response to changes in the demand for assimilates. The authors of [22] showed that whole-ear photosynthesis correlated better with flag leaf photosynthesis. Our research was a comparative study on the effects of Fusarium head blight disease on the photosynthetic process and the agronomical and quality traits in three winter wheat varieties with different resistances to FHB. We checked whether the photosynthesis in flag leaves can maintain a standard quality performance under FHB stress in the heads. FHB symptoms, in terms of bleaching spikelets, were visible 7–11 days after inoculation. Photosynthetic parameters of flag leaves obtained by chlorophyll *a* fluorescence measurements, as indicators of photosynthetic functions in plant, as well as agronomical and quality traits, were determined in a control group and an FHB treatment group. It is important to note that most previous studies on photosynthesis were performed in a controlled environment, so they may not fully reveal real field conditions. In our research, the photosynthetic parameters of flag leaves are presented, along with agronomical traits (1000 kernel weight is yield component) and the composition and quantity of wheat proteins, the main determinants of the technological properties of wheat dough. According to Yang et al. (2016) [10], 1000 kernel weight can be useful for determining the response of different varieties to disease infection.

‘Vulkan’ had a non-significantly lower reduction in test weight under Fusarium infection in comparison to the control, and inoculated ‘Golubica’ plants, compared to the control group, saw a sharply reduced test weight. The 1000 kernel weight parameter was at the same level in all three varieties in both treatments. Although FHB-resistant ‘Vulkan’ had lower photosynthetic parameter values, this variety maintained a high yield in the inoculated treatment group in comparison to the control group (data not shown), but, compared to ‘Kraljica’ and ‘Golubica’, it had the lowest % of HMW glutenin subunits in the control group. This was expected because grain yield and quality parameters are traits that are negatively correlated [23], but they are also the most important quantitative traits of winter wheat and a priority of wheat breeders [24].

In normal conditions, ‘Golubica’ has an optimal proportion of HMW subunits [17]; however, in our study, ‘Golubica’ had a significantly lower % of GLU, % of HMW subunits, and % of LMW subunits in the FHB treatment group. A similar decline in wheat quality, due to an increase in intensity of *Fusarium* spp. Contamination, has been observed by Papoušková et al. [25]. HMW subunits were most affected in ‘Kraljica’ and ‘Golubica’.

Assimilates transported to the grain during grain filling in wheat are mainly provided by three sources: (i) flag leaf photosynthesis; (ii) pre-anthesis reserves; and (iii) ear photosynthesis [26]. Additionally, it is often considered that the grain yield of wheat is limited by the strength of the sink rather than by the availability of assimilates [27], but this is not yet clear for wheat grain quality. Statistically significant differences were found in the F_v_/F_m_ and PI parameters among all tested varieties at different measurement times, and ‘Vulkan’ was different from the two other varieties, with the lowest PI and F_v_/F_m_ values. This varietal difference could be explained by the genetic basis of photosynthetic traits within varieties [28] as well as the variation in leaf senescence induction, which occurs later [14] and may also diminish photosynthetic performance. However, ‘Vulkan’, in comparison to ‘Golubica’ and ‘Kraljica’, was less affected by FHB infection in terms of disease symptoms. In general, there were no significant differences in PI and F_v_/F_m_ between the two treatments. We conclude that FHB does not have strong effects on PI and F_v_/F_m_, namely, the photosynthesis in the leaves. This is in accordance with research by Yang et al. (2016) [10]. The lowest value of PI in ‘Vulkan’, in comparison to ‘Golubica’ and ‘Kraljica’, may imply that, despite Fusarium infection, ‘Vulkan’ can maintain a dynamical balance of photosynthetic products between photosynthetic sources (leaves) and nonphotosynthetic sinks (developing seed) [10]. This potential ability of ‘Vulkan’ may be the basis of its reduced sensitivity to FHB. Alterations of such dynamical source/sink balance, seen as enhancement of photosynthetic-related parameters in ‘Kraljica’ and ‘Golubica’, imply an inefficient flow of energy-rich molecules produced in photosynthesis to the developing seed. The PI and F_v_/F_m_ values indicate a lower photosynthetic performance at first measurement in both treatments in all three varieties, but the values of PI and F_v_/F_m_ increased until 4 days after inoculation. At 7 days after inoculation, generally, significant decreases in those parameters were observed in all three varieties in both treatments (except PI for ‘Kraljica’ in the control group), suggesting a downregulation of photosystem II, which may indicate an initiation of leaf senescence [14,28]. Additionally, an induction of grain filling and an onset of senescence in wheat are concurring processes. It is known that the disassembly of a photosynthetic apparatus is a major event in senescence, resulting in increased nitrogen content originating from photosynthetically active cells in chloroplast, mainly as Rubisco degradation residues [29]. However, previous studies have suggested that Rubisco activity is not the main photosynthesis limiting factor and that the Rubisco degradation process activates persisting PSII centers to work highly efficiently to prevent and control breakdown process. In both treatments, ‘Kraljica’ and ‘Golubica’, as compared to ‘Vulkan’, which had the lowest % of HMW subunits in the control group and the lowest PI value in both treatments, showed a better % of HMW subunits as well as a higher photochemical quantum yield of PSII (F_v_/F_m_) in the last measurement. We can conclude that photosynthesis of the ear was partly inhibited, which led to significant losses in certain agronomical and quality traits in ‘Kraljica’ and ‘Golubica’. The low PI in ‘Vulkan’ could be a result of low head infection; this variety thus would not need to intensify the photosynthesis in flag leaves. This variety might have had enough fructan accumulates in the stem internodes and leaf sheaths, which were thus remobilised during the later stages of grain filling, with no losses in the FHB treatment group, in comparison to the control group, in % of AG , % of GLI, % of GLU, % of HMW subunits, % of LMW subunits, test weight, or 1000 kernel weight.

FHB did not have a significant effect on fluorescence parameters in flag leaves in the FHB-resistant variety (‘Vulkan’) compared with the FHB-susceptible variety (‘Golubica’); however, it did have a great impact on the yield components in the susceptible variety, which is in accordance with an investigation by Živčák et al. (2008) [9]. Thus, we conclude that the essential physiological process that remobilizes nutrients for grain production has failed due to strong disease severity in the heads of ‘Golubica’. Our findings suggest that FHB has a negative effect on test weight in FHB-susceptible varieties and on glutenins in FHB-susceptible varieties and moderately resistant varieties, but no significant effect on photosynthetic parameters. The advantage of such complex analysis lies in the fact that it can indicate stress in plants even before visible symptoms appear on the leaves [30]. The next step should be an investigation of changes related to photosynthetic parameters in wheat ears for the detection of biotic stress. Still, however, the photosynthetic contribution to grain filling and quality is not clear, and more complex studies of this type should be conducted, where the photosynthetic contribution in ears and leaves to wheat quality grain is simultaneously measured in both organs.

## 4. Materials and Methods

### 4.1. Inoculum Production

The pathogen inoculum consisted of two different *Fusarium* species. To produce macroconidia of *F. culmorum* (Wm.G.Sm.) Sacc., a mixture of wheat and oat grains (3:1 by volume) was soaked in water overnight in 250 mL glass bottles [31]. Water was decanted and seeds were autoclaved. After seeding with the *Fusarium* strain, the seeds were kept for 2 weeks at 25 °C in the dark and thereafter incubated in the refrigerator for 3 weeks. Conidia were washed from the kernels and the concentration of the conidial suspension was set to 1 × 10^5^ mL^−1^. Inoculum with *F. graminearum* Schwabe was prepared with the bubble breeding method using a liquid mung bean medium [32]. A final concentration of the conidial suspension of *F. graminearum* inoculum was set to 1 × 10^5^ mL^−1^. The spore suspensions were set to a concentration so that a single bottle of one strain contained a sufficient amount of suspension (>900 mL), which could be diluted in 100 L of water right before inoculation (100 mL per m^2^). The aggressiveness test was done in Petri dishes as described by Lemmens et al. [33] (data not shown).

### 4.2. Field Trial

Three wheat varieties were used for FHB resistance testing in 2015/2016 at the experimental field of Agricultural Institute Osijek, Croatia (45°32′ N, 18°44′ E). Those three genotypes originated from the Agricultural Institute Osijek. ‘Vulkan’ is a bread wheat with high yield, moderate quality, and early heading, earlier characterized as FHB-resistant [34], while ‘Kraljica’ has good quality and high yield with lodging resistance. ‘Golubica’ is a high quality variety with moderate yield, previously characterized as Fusarium-susceptible [35]. The soil type was eutric cambisol. The average annual precipitation in the growing season was 595 mm and the average annual temperature was 9.73 °C. Varieties were sown in eight row plots with a 7 m length and a 1.08 m width in October at a sowing rate of 330 seeds m^−2^, where treatments (Fusarium and control treatments) were replicated in two plots. Spray inoculations with *Fusarium* spp. were performed individually for each genotype at flowering (Zadok’s scale 65) [36] using a tractor-back sprayer. To maintain moisture, ears were water sprayed with tractor back-sprayer on several occasions during the day. General resistance (percentage of diseased spikelets in the plot) was estimated according to a linear scale (0–100%) 26 days after inoculation.

### 4.3. Chlorophyll a Fluorescence

Ten leaves of each treatment (from both the control plants and the Fusarium-stressed plants) were analyzed at different times after wheat flowering (at 1, 2, 4, 7, and 14 days after Fusarium inoculation). Chlorophyll *a* fluorescence of flag leaves was measured by a Plant Efficiency Analyser (Handy PEA, Hansatech, Norfolk, UK) in the morning hours (07:00–09:00 h) in order to bring out certain biophysical parameters of PSII functioning calculated by the JIP test. After the adaptation of leaves to darkness, a single 1 s light pulse (3500 μmol m^−2^ s^−1^) was applied with the help of three light-emitting diodes (650 nm). The maximum quantum yield (efficiency) of PS II photochemistry (F_v_/F_m_) and the performance index were calculated according to the equations reviewed by Stirbet and Govindjee [37].

### 4.4. Proteins Characterization

The wheat protein extraction from 100 mg of flour sample was done stepwise accordingly to the procedure of Wieser et al. (1998) [31]. Proteins separation was carried out using Perkin Elmer LC 200 chromatograph controlled by Total-Chrom software (Perkin Elmer Instruments, Waltham, MA, USA) on a Discovery Bio Wide Pore C18 column (300 Å pore size, 5 μm particle size, 4.6 × 150 mm i.d.) (Sigma-Aldrich Chemie GmbH, Taufkirchen, Germany). Amounts of 0.1% trifluoroacetic acid (TFA) in water (v/v) and 0.1% TFA in acetonitrile (ACN) were applied as mobile phase and 20 μL samples were injected for analyses. AG, GLI, and GLU fractions were eluted with a linear gradient from 24 to 58% ACN over 30 min at a 1 mL min^−1^ flow using a column temperature of 50 °C. All determinations were made in duplicate. The peak areas under AG, GLI, and GLU chromatograms were summed and used as a direct measure of total content of extractable wheat proteins. Consequently, the proportions (%) of protein fractions and single protein types were calculated [38].

### 4.5. Statistical Analysis

Statistical analysis was done using analysis of variance (ANOVA) followed by the Fisher’s LSD test (α = 0.05) by Statistica version 12.0 (Statsoft Inc., Tulsa, OK, USA). The reported data for fluorescence and proteins parameters represent the mean ± standard error (SE).

## Figures and Tables

**Table 1:** ANOVA for agronomical and quality traits among three varieties in two different treatments.

Source of Variation	Df	Mean Square						
		TW	TKW	AG	GLI	GLU	HMW	LMW
Variety	2	102.6 ***	14.7 **	2.810 *	43.40 ***	28.87 ***	18.11 ***	30.29 ***
Treatment	1	13.44 **	11.21 *	0.090ns	21.12 ***	18.45 ***	2.98 ***	9.11 ***
Variety * Treatment	2	73.86 ***	21.05 *	6.741 ***	24.94 ***	32.41 ***	2.93 ***	13.13 ***
Error	6	1.17	1.39	0.364	0.3	0.32	0.09	0.3

Significant differences were calculated according to Fishers’ LSD test at *p* < 0.001. ***,**,* = significant at *p* < 0.001, 0.01 and 0.05, respectively. AG: albumins; DF: degrees of freedom; GLI: gliadins; GLU: glutenins; HMW: high-molecular-weight glutenins subunits; LMW: low-molecular-weight glutenins subunits; TKW: thousand kernel weight; TW: test weight.

**Table 2:** ANOVA for PI and F_v_/F_m_ among three varieties in two different treatments at five measuring points.

Source of Variation	Df	Mean Square	
		PI	F_v_/F_m_
Variety	2	27.096 ***	0.0048 **
Measurement time	4	44.415 ***	0.0246 ***
Treatment	1	0.052	0.0001
Variety × Measurement time	8	2.894 ***	0.0005
Variety × Treatment	2	0.931	0.0005
Measurement time × Treatment	4	0.368	0.0006
Variety × Measurement time × Treatment	8	0.157	0.0002
Error	561	0.583	0.0003

Significant differences were calculated according to Fishers’ LSD test at *p* < 0.001. ***,** = significant at *p* < 0.001, 0.01, respectively. DF: degrees of freedom; F_v_/F_m_: maximum quantum yield of PSII photochemistry; PI: performance index.

**Table 3:** Statistical differences between varieties under the same treatment for average values of all measurement times.

	PI		F_v_/F_m_	
	Con	FHB	Con	FHB
Vulkan	3.5330 ^b^	3.4145 ^b^	0.8275 ^b^	0.8250 ^b^
Kraljica	4.0642 ^a^	4.2181 ^a^	0.8331 ^a^	0.8366 ^a^
Golubica	4.0890 ^a^	4.1230 ^a^	0.8344 ^a^	0.8357 ^a^

Letters (a,b) indicate significantly different values (*p* < 0.05) among different varieties under the same treatment. Con: control treatment; FHB: Fusarium Head Blight treatment; F_v_/F_m_: maximum quantum yield of PSII photochemistry; PI: performance index.
